# Caffeic acid phenethyl ester decreases acute pneumonitis after irradiation *in vitro *and *in vivo*

**DOI:** 10.1186/1471-2407-5-158

**Published:** 2005-12-09

**Authors:** Miao-Fen Chen, Peter C Keng, Paul-Yang Lin, Cheng-Ta Yang, Shuen-Kuei Liao, Wen-Cheng Chen

**Affiliations:** 1 Department of Radiation Oncology, Chang Gung Memorial Hospital, Chia-Yi, Taiwan; 2 Graduate Institute of Clinical Medical Science, Chang Gung University, Toyuan, Taiwan; 3 Department of Radiation Oncology, Biochemistry and Biophysics, University of Rochester School of Medicine and Dentistry, Rochester, New York, USA; 4 Department of Pathology, Chang Gung Memorial Hospital, Chia-Yi, Taiwan; 5 Department of Internal medicine, Chang Gung Memorial Hospital, Chia-Yi, Taiwan

## Abstract

**Background:**

Lung cancer is relatively resistant to radiation treatment and radiation pneumonitis is a major obstacle to increasing the radiation dose. We previously showed that Caffeic acid phenethyl ester (CAPE) induces apoptosis and increases radiosensitivity in lung cancer. To determine whether CAPE, an antioxidant and an inhibitor of NF-kappa B, could be a useful adjuvant agent for lung cancer treatment, we examine the effects of CAPE on irradiated normal lung tissue in this study.

**Methods:**

We compared the effects of CAPE on cytotoxicity and intracellular oxidative stress in normal lung fibroblast and a lung cancer cell line. For *in vivo *analysis, whole thorax radiation (single dose 10 Gy and 20 Gy) was delivered to BALB/c male mice with or without CAPE pretreatment. NF- kappaB activation and the expression levels of acute inflammatory cytokines were evaluated in mice after irradiation.

**Results:**

The *in vitro *studies showed that CAPE cause no significant cytotoxicity in normal lung as compared to lung cancer cells. This is probably due to the differential effect on the expression of NF-kappa B between normal and malignant lung cells. The results from *in vivo *study showed that CAPE treatment decreased the expression of inflammatory cytokines including IL-1 alpha and beta, IL-6, TNF-alpha and TGF- beta, after irradiation. Moreover, histological and immunochemical data revealed that CAPE decreased radiation- induced interstitial pneumonitis and TGF-beta expression.

**Conclusion:**

This study suggests that CAPE decreases the cascade of inflammatory responses induced by thoracic irradiation without causing toxicity in normal lung tissue. This provides a rationale for combining CAPE and thoracic radiotherapy for lung cancer treatment in further clinical studies.

## Background

Lung cancer is the leading cause of cancer death worldwide. Radiotherapy is an important modality of cancer treatment. Because radiation pneumonitis is a major obstacle to increasing the radiation dose, it is important to determine how the incidence of radiation- induced complication might be decreased and how the dose that normal lung can tolerate might be increased. Caffeic acid phenethyl ester (CAPE) is a phenolic antioxidant, an active anti-inflammatory component of propolis [1-3]. Several studies and our previous study have shown that the compound elicits several interesting biological functions including inducing apoptosis in various tumor cell types and anti-inflammatory properties [4-6]. Since reactive oxygen species (ROS) is the major mediators for radiation induced damage [7], a treatment combining radiation with an antioxidant might provide a strategy for preventing radiation injury to normal tissues [8]. Various investigators have demonstrated that radiation-induced proinflammatory cytokines contributed significant complications associated with radiotherapy [9,10]. Early manipulation of inflammatory responses could be useful in modifying subsequent late effect [14]. CAPE is an active anti-inflammatory compound [11,12], and a specific inhibitor of the transcription factor nuclear factor-κB (NF-κB) [13,14]. It might play a role in protecting normal tissue against damage from radiation treatment. However, the actual effects of CAPE on irradiated normal lung and the underlying mechanisms of protection are still unclear. An initial goal of our study was to assess the capacity of CAPE to decrease radiation pneumonitis. We utilized two cell lines, normal lung fibroblast (WI-38) and lung cancer cells (A549), to compare the effects of CAPE on normal and malignant lung cells in the presence and absence of radiation treatments in vitro. We were specifically interested in evaluating the effects of CAPE on the intracellular ROS and NF-κB activation in these cells. Another important goal of this study was to evaluate the efficiency of CAPE in an animal model of radiation- induced pneumonitis.

## Methods

Human lung cancer A549 cell and human normal lung fibroblast WI-38 cells were obtained from ATCC. They were routinely cultured in complete MEM medium and maintained in a 37°C incubator with 5% CO_2 _and 95% air.

### Cell growth curve analysis with radiation

Exponentially growing cells were treated with or without CAPE 6 μg/ml for 1 h prior irradiation. Cells were irradiated (9 Gy in a single fraction) with a 6 MeV electron beam generated by a linear accelerator at a dose rate of 300 cGy/min. After irradiation, the cells were allowed to grow in the incubator. Viable cells were counted on day 2, 4, 6 and 8 after treatment.

### Intracellular H_2_O_2 _and glutathione (GSH) analysis

Intracellular H_2_O_2 _was assayed with a fluoresence dye, DCFH-DA, using a FACS caliber flow cytometery, as described previously [15]. To determine the intracellular GSH, we performed a colorimetric assay using ApoAlert glutathione detection kit (Clontech, CA). Cells were lysed and monochlorobimane was added to the lysate at 37°C for at least 15 min. The fluorescence intensity was measured in a plate reader at 395 nm.

### Electrophoretic mobility gel shift assays to analyze the binding activity of NF-κB

The cells were treated with nuclear extraction reagent 4 hour after 9 Gy irradiation (Pierce, Rockford, IL). The nuclear proteins from BALB/c mice lung tissues were collected 12 hours after a 20 Gy irradiation. Four murine lung tissues from each group were checked. The protein content was measured by the Bradford method. The DNA oligonucleotides used for NF-κB binding were 5'-TTGTTACAAGGGACT TTCCG TGGGGACTTTCC AGGGAGGC GTGG-3' for human and 5'- AGTTGAGGGACTT TCCCAGGC-3' for mouse. Nuclear extracts were incubated with the biotin labeled DNA probe for 20 min at room temperature. The DNA- protein complex was separated from free oligonucleotide on a 5% polyacrylamide gel. After electrophoresis, the DNA-protein complex was transferred to a nylon membrane and cross-linked with UV. The membrane was incubated with streptavidin- horseradish peroxidase-conjugate and detected by ECL (Pierce, Rockford, IL).

### Mice, radiation and CAPE treatment

Male BABL/c mice between 6 and 8 weeks old were purchased from the Animal Center of the National Science Council, Taipei, Taiwan. The protocol of animal experimentation was approved by the Chang Gung Memorial Hospital Experimental Animal Committee. For lung irradiation, anesthetized mice were restrained in modified Perspex tubes. The whole thorax was irradiated by 6 MV X-ray from a linear accelerator and a 1.5 cm bolus on the surface. Control mice were subjected to sham-irradiation. The mice were divided into four groups: control, CAPE alone, irradiation, irradiation with CAPE treatment. To analyze NF-κB activation and the expression of inflammatory cytokines, 24 mice were irradiated with 20 Gy and sacrificed at the indicated times. For histological examination, 12 mice received 10 Gy irradiation. The mice were injected intraperitoneally with CAPE (10 mg/kg, solubilized in saline containing 20% Tween 20) 30 min before irradiation and once a day for 10 days after irradiation.

### The expressions of inflammatory cytokines analyzed by RNase protection assay (RPA) and real time RT-PCR

The mice were killed 6 hours and 24 hours after 20 Gy irradiation and RNA was extracted. Four mice from each group were sacrified at the indicated times and the lungs were dissected. Total cellular RNA was isolated using Trizol (Gibco, Grand Island, NY) reagents for RPA and real time RT PCR. Two probe sets-mck2b and mck3b (Pharmingen, San Diego, CA) were used for RPA. The biotin-labeled probes (Ambion, Austin, TX) were hybridized to the target mRNA. The samples were mixed with loading buffer and separated on a 9% sequencing gel by electrophoresis. The intensity of each gene was normalized against with L32 and GAPDH expression. Two μg RNA was subjected to reverse transcription using the superscript II kit (Invitrogen, Carlsbad, CA) with random primer to obtain the first cDNA strand. Primers for IL-1 α/β, IL-6, TNF-α, TGF-β were used for PCR analysis (Applied Biosystems, Foster, CA). To allow for loading differences, a GAPDH primer was used as control. Optimized PCR was performed on an iCycler iQ multicolor real time PCR detection system. Significant PCR fluorescent signals were normalized to a PCR fluorescent signal obtained from the mean value for sham-irradiated control mice.

### Histologic and immunochemical staining analysis for pulmonary inflammation

Treated and control mice were sacrificed by cervical dislocation at 3 and 12 weeks after 10 Gy irradiation. Three mice for each group were used. The whole lungs were perfused via the trachea before they were removed; they were fixed in formalin after removal. For histologic analysis, the lobes were fixed in 10% buffered formalin; paraffin-embedded and sectioned at an average thickness of 5 μm. The mounted sections were subjected to H&E and immunochemical staining. They were incubated overnight with goat anti- mouse TGF-β1antibody (Santa Cruz, CA) After three wash with PBS, the sections were incubated with biotinylated anti-goat IgG followed by peroxidase-avidin staining, washed again in PBS, and treated with 3-amino-9 ethylcarbazole solution as a chromogen. The specimens were counterstaining with hematoxylin. Omission of the primary antibody was used as a negative control, and murine bowel tissue was used as a positive control for the presence of TGF-β1.

## Results

### CAPE causes no cytotoxicity or radiosensitization in normal lung cells

**Figure 1:**
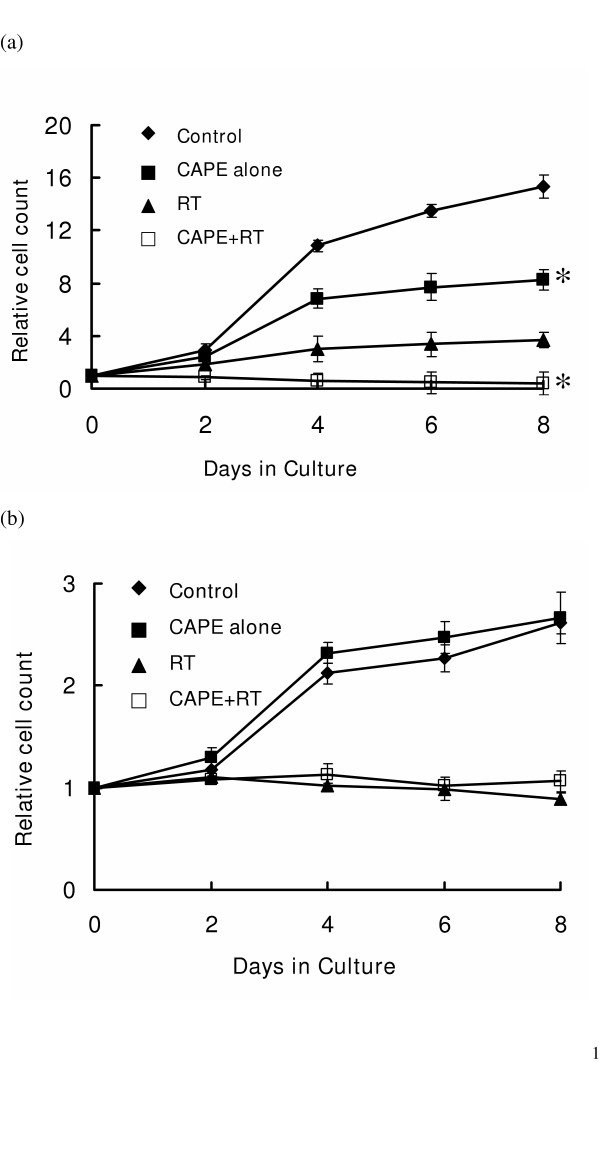
The growth curves of A549 lung cancer cells and WI-38 cells in the presence of CAPE and irradiation. Exponentially growing cells were treated with or without 6 μg/ml CAPE for 1 h prior to irradiation. Viable cells were counted on day 2, 4, 6 and 8 after treatment. The growth curves were obtained by plotting the number of viable cells as a function of time in culture for 8 days. (a) Growth curves of A549 cancer cells; (b) Growth curves of WI-38 cells. CAPE significantly reduces *in vitro *cell growth of A549 with or without irradiation, while no inhibition of growth was detected in WI-38 cells. Three independent experiments were performed for these curves. Y axis represents the relative cell count, normalized to the cell numbers on Day 0. * P < 0.05, day 4, 6 and 8, CAPE treated cells verse CAPE- untreated cells.

In our previous report, we demonstrated that 6 μg/ml CAPE caused significant cytotoxicity and increased apoptosis in lung cancer cells [5]. However, the percentage apoptosis showed no obvious increase in WI-38 after CAPE treatment (data not shown). To compare the effects of CAPE in lung cancer and normal lung cells, we treated the cells with 6 μg/ml CAPE for 1 hour prior to radiation and evaluated the cytotoxicity and radiosensitizing effects. As shown in Figure 1, CAPE had no cytotoxicity and radiosensitization effects on WI-38 normal lung cells, in contrast to A549 lung cancer cells.

**Figure 2:**
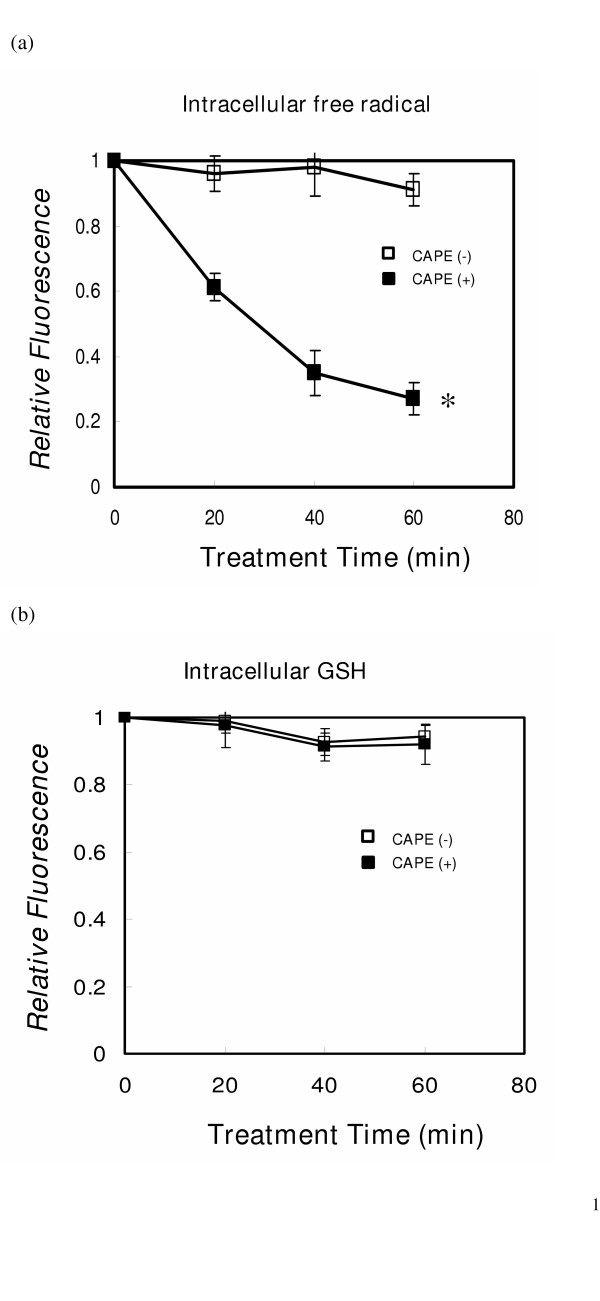
Intracellular peroxide and glutathione content of WI-38 cells. Determination of peroxides and GSH in intact cells (WI-38 control cells or cell treated with CAPE) was accomplished by fluorescence measurement as described in the Materials and Methods section. (a) Intracellular H_2_O_2 _levels. (b) intracellular GSH levels. Data are presented as the mean ± SE of three separate experiments. Y axis represents the relative fluorescence, normalized to the fluorescence value of control cells. * P < 0.05, CAPE treated cells verse CAPE- untreated cells.

The CAPE- induced decrease of intracellular GSH described in our previous study might have contributed the radiosensitization effect on A549. To explore the possible mechanisms responsible for differential radiosensitization in A549 and WI-38, the intracellular H_2_O_2 _and GSH were measured after CAPE treatment. The antioxidant effect of CAPE decreased the intracellular H_2_O_2 _within 1 h in WI-38 cells (Fig. 2A), as found in A549 cells in our previous study. However, CAPE did not decrease the intracellular GSH level in WI-38 cells as it does in A549 lung cancer cells (Fig. 2B). This differential effect could explain, at least in part, why CAPE- induced radiosensitization is found in A549 but not WI-38.

### CAPE attenuates NF-κB expressiondifferentially in normal lung and lung cancer cells

**Figure 3:**
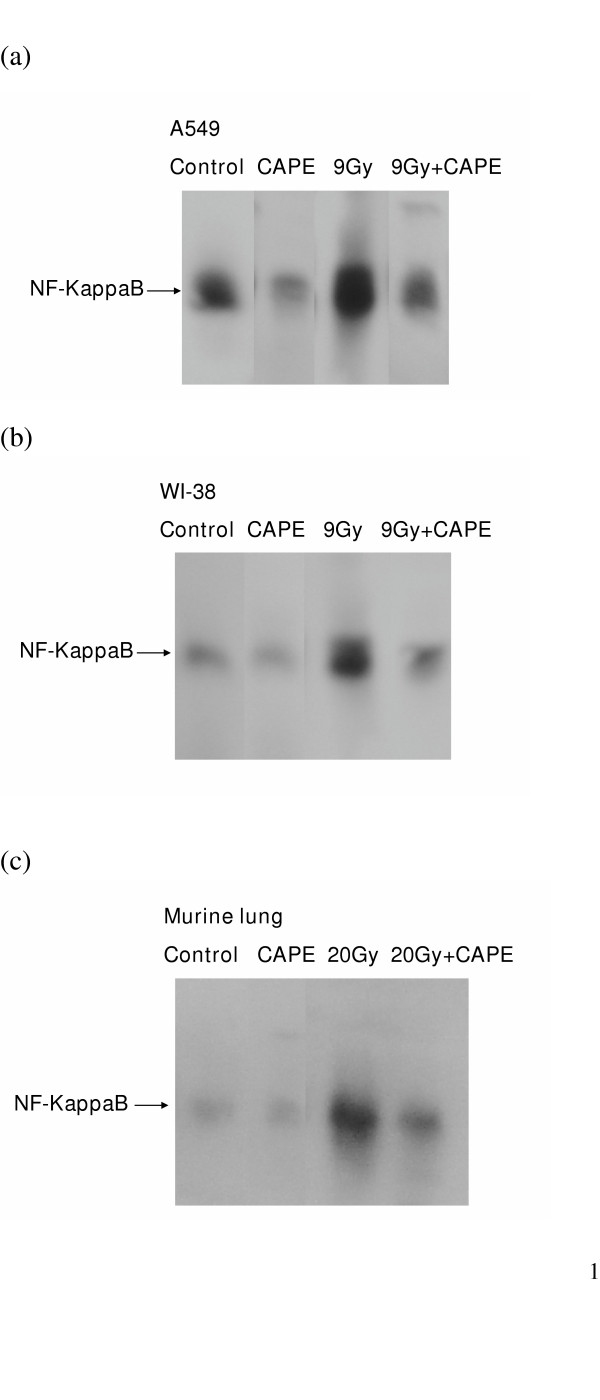
Inhibition of irradiation- induced activation of DNA binding of NF-κB by CAPE *in vitro *and *in vivo*. Nuclear extracts were isolated and mobility gel shift assays were performed as described in the Material and Methods Section. Representative figures are ahown for (a) A549 cancer cells; (b) WI-38 cells 4 hours after 9 Gy irradiation; and (c) normal mice lung 12 hours after 20 Gy irradiation. These demonstrate the binding activity of nuclear NF-κB is higher in A549 cancer cells. CAPE inhibited the binding activity of NF-κB in A549 cancer cells with or without irradiation and in normal lung cells after irradiation.

CAPE is a specific inhibitor of NF- κB that works by inhibiting the interaction of the transcription factor with DNA. The putative target genes of NF- κB are mainly involved in immune and inflammatory responses. To demonstrate the activation of NF- κB after CAPE treatment, gel shift experiments were conducted in both cell lines (A549 lung cancer cell line and WI-38 normal lung cell) and in mouse lung tissue following various treatments. As shown in Figure 3A and 3B, the nuclear binding of NF-κB is higher in lung cancer cells than in normal lung cells, and 6 μg/ml CAPE treatment significantly decreased binding in lung cancer cells as compared with normal lung cells. Irradiation increased the nuclear binding of NF-κB in both lung cancer cells and normal lung cells. Pre-treatment with 6 μg/ml CAPE for 1 h decreased the augmentation of NF-κB binding activity by irradiation in both cell types. Figure 3C demonstrates the similarity between the nuclear proteins obtained from murine lung tissues and WI-38. There was not change in NF-κB binding in unirradiated lung tissues after CAPE treatment. Irradiation with 20 Gy promoted NF-κB binding, and treatment with CAPE attenuated this effect 12 h after irradiation. The results clearly showed that irradiation activates NF- κB binding and the activation is attenuated by CAPE.

### Effect of CAPE on the radiation- induced expression of proinflammatory cytokines as revealed by RPA and real- time PCR

**Figure 4:**
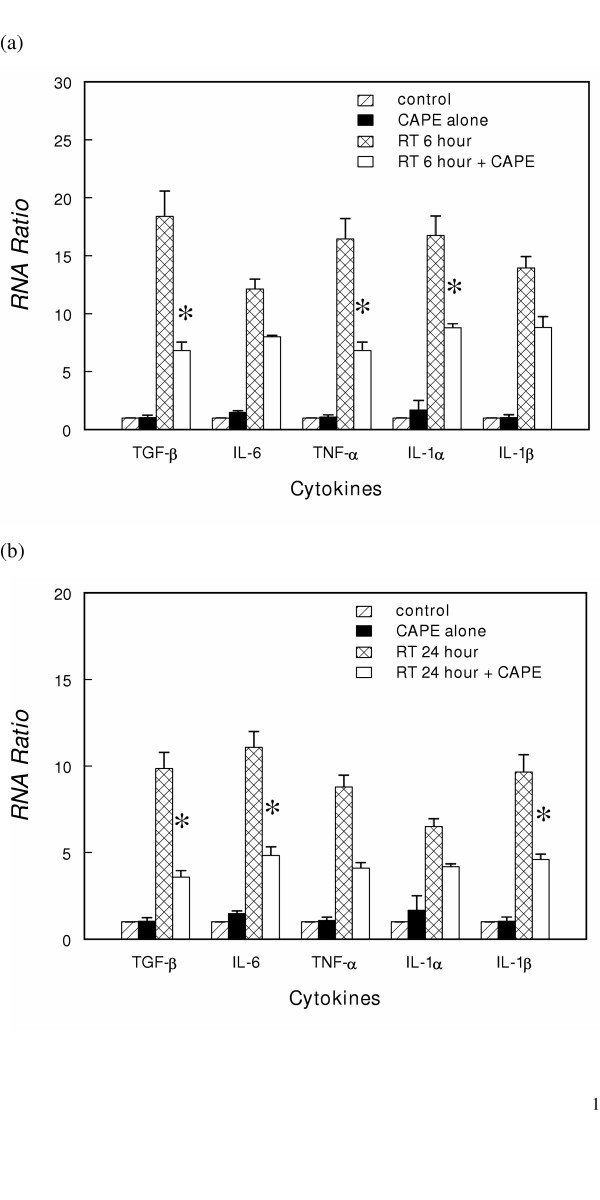
Effects of CAPE treatment on radiation-induced increases in inflammatory cytokine mRNA levels *in vivo*. The TGF-β, TNF-α, IL-1α/βand IL-6 mRNA levels (a) 6 hours (b) 24 hours after20 Gy irradiation. The results are expressed as RNA ratio. The RNA ratio was determined from the mRNA level at indicated time after treatment to control for a specific gene. Data are the mean ± SE. * *P *< 0.05, CAPE-treated irradiated mice versus CAPE-untreated irradiated mice.

We used a non-radioactive ribonuclease protection assay to screen the expressions of various cytokines mRNAs. The mRNAs detected were for cytokines involved in acute inflammation and the fibrosis of pneumonitis: TNF-α/β, IL-6, IFN-β/γ, IL-6, IL-10, IL-1α/β, IL12 and MIF. These cytokine mRNAs were barely detectable in control but the levels were elevated after 20 Gy pulmonary irradiation. After combined radiation and CAPE treatment, there was a decrease in the trend towards overexpression of TNF-α, IL-6, IL-1α/β and TGF-β mRNA (data not show). Furthermore, we quantified the expression of these cytokines after radiation and CAPE treatment by real-time RT-PCR. Low expression levels were noted in unirradiated BALB/c mice and there were no obvious changes after 10 mg/kg CAPE treatment. Irradiation (20 Gy) induced a significant increase in TNF-α, IL-6, IL-1α/β and TGF-β mRNA 6 hours and 24 hours after irradiation (Fig. 4A and 4B). The attenuating effect of CAPE measured by RPA was quantified by real-time PCR. At 6 hours, CAPE treatment reduced the increase in the levels of the TNF-α, IL-1α and TGF-β mRNA by half (p < 0.05 verse irradiated untreated mice), and IL-6 and IL-1β mRNA by one quarter. The inhibitory effect of CAPE on IL-6 and IL-1β mRNA levels were more significant 24 h after irradiation (p < 0.05 verse irradiated untreated mice).

### CAPE attenuates the induction of pulmonary inflammation by irradiation

**Figure 5:**
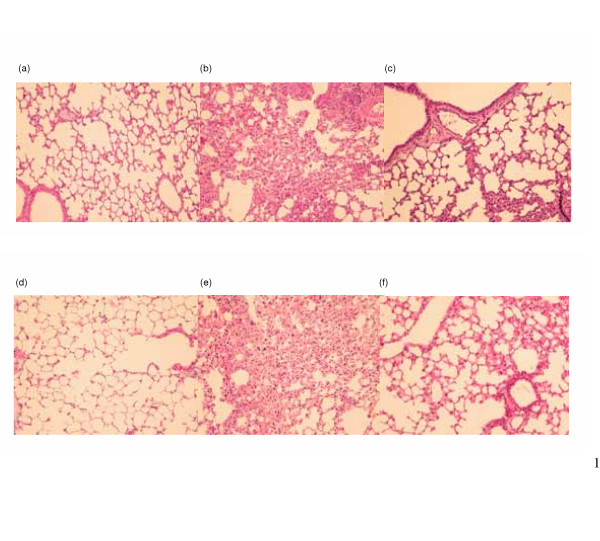
Histologic analyses with H&E staining on irradiated murine lung tissue. Three mice from each group were checked. Representative slides are shown for (a) unirradiated control mice at 3 weeks after sham-irradiation; (b) untreated mice at 3 weeks after 10 Gy irradiation; (c) CAPE-treated mice at 3 week after irradiation 10 Gy irradiation; (d) unirradiated control mice at 12 weeks after sham-irradiation; (e) untreated mice at 12 weeks after 10 Gy irradiation; (f) CAPE-treated mice at 12 week after irradiation 10 Gy irradiation. These demonstrate that interstitial pneumonia with increased acute inflammatory infiltrate in the interstitium was detected at 3 weeks and 12 weeks after irradiation. CAPE treatment attenuated the extent of inflammation. Magnification × 200

No lesions were observed in non-irradiated lung from control mice. Microscopic examination of the lungs 3 weeks after 10 Gy irradiation revealed an increase in acute inflammatory infiltrate in the interstitium. After CAPE treatment, the degree of interstitial pneumonitis 3 weeks after 10 Gy irradiation was less pronounced (Fig 5A–C). Similar changes persisted 12 weeks after irradiation (Fig 5D–F).

**Figure 6:**
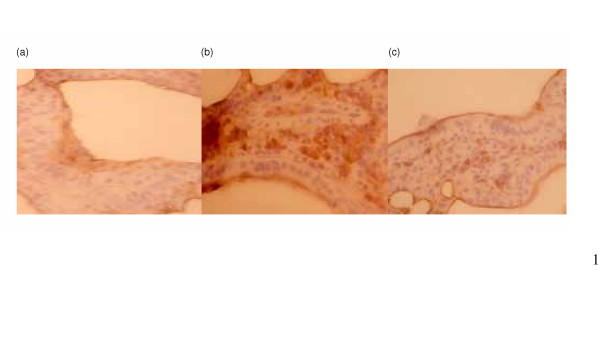
Immunohistochemical staining with TGF-β antibody on murine lung tissues. Three mice from each group were checked. Representative slides are shown for (a) unirradiated control mice at 3 weeks after sham-irradiation; (b) untreated mice at 3 weeks after 10 Gy irradiation; (c) CAPE-treated mice at 3 week after irradiation 10 Gy irradiation. These demonstrated that CAPE treatment attenuated the increased TGF-β immunoreactivity after irradiation. Magnification × 250.

Immunochemical analysis showed that unirradiated lung tissue exhibited very low TGF-β1 immunoreactivity in the parenchyma and muscularis propria. By 3 weeks after 10 Gy irradiation, positive TGF-β1 staining had increased. CAPE treatment attenuated the radiation- induced increase. (Fig 6)

## Discussion

In this study, CAPE cause no significant cytotoxicity and radiosensitization in normal lung cell, in contrast to that noted in lung cancer cells. CAPE is a potent and specific inhibitor of activation of nuclear transcription factor NF-κB [12]. NF-κB activation could induce compensated mechanisms and decrease apoptosis. The inhibition of NF-κB by CAPE is a possible mechanism for tumor cell cytotoxicity [16-18]. We found significant higher expression of NF-κB binding in lung cancer cells than normal lung cells. CAPE significantly decreased the NF-κB binding activity in lung cancer cells. In contrast, it caused no significant change in the expression of NF-κB binding in normal lung. The differential downregulation of NF-κB by CAPE might explain why CAPE caused significant cytotoxicity and growth inhibition in lung cancer cells but not normal lung cells. Furthermore, we found that CAPE had a radiosensitizating effect on lung cancer cells but not on normal lung cells. The mechanism underpinning this radiosensitization may be related to intracellular ROS, GSH and NF-κB [19-21] and it needs further investigation. Intracellular GSH might play a role because we noted that GSH levels decrease after CAPE treatment in tumor cells but not in normal lung cells. GSH decreases radiation-induced damage through its function as a free radical scavenger. A high concentration of intracellular thiol is an important way to resist cytotoxic and radiation damage in cancer cells. The depletion of GSH in lung cancer cells is consisting with the differential radiosensitization between tumor cells and normal lung cells.

The lung is the major dose-limiting organ for radiotherapy in thoracic region and radiation pneumonitis is a serious complication of lung cancer treatment by radiotherapy. Acute pneumonitis is characterized by edema, infiltration of inflammatory cells and thickening of the alveolar septa. Late radiation- induced lung damage is characterized by pulmonary fibrosis, which is usually proceeded by fibrosing alveolitis [22]. Franko et al [23] pointed out the pathomorphological effects in irradiated lung in relation to the lung function; fibrosis was first observed 8 weeks post-irradiation at 10.3 Gy and the number of fibrotic lesions had increased 10- fold by 14 weeks. The response induced by radiation *in vivo *is associated with increased expression and activity of inflammatory cytokines. NF-κB is believed to play a pivotal role in the induction of cytokine expression in inflammatory response [24,25]. Haase et al [26] reported that DNA binding by NF-κB is activated for 6 months after irradiation of the rat lung. This might play a role in sustaining chronic inflammation and hyerproliferation of mesenchymal cells after radiation. NF-κB plays a key role in the induction of these cytokines *in vivo*. The efficacy of CAPE in inhibiting NF-κB activation and proinflammatory production has been demonstrated [13,14,27,28]. Fitzpatrick et al [14] reported that CAPE significantly attenuated bacterial peptidoglycan polysaccharise-induced colitis and reduced the inflammatory cytokine level. Linard et al [27] demonstrated that CAPE treatment reduced the NF-κB activation and attenuated the increase in IL-6 and IL-1β expression in intestine. However, there were few reports about the relationship between CAPE and radiation pneumontits. The present study has demonstrated that CAPE treatment inhibits NF-κB activation and reduces the overexpression of genes involved in the acute inflammatory response, including IL-1α/β, TNF-α and IL-6 after irradiation in the mouse lungs. We found that CAPE treatment inhibited NF-κB activation and reduced the overexpression of genes involved in the acute inflammatory response including IL-6 and IL-1β, consistent with the results of Linard's series. In addition, the finding of the inhibition of TNF-α and IL-1 by the selective blockade of NF-κB pathway is similar to observations reported in alveolar epithelial cells [25,29]. Because these elevated cytokines are closely related to radiation induced pneumonitis [30,31], this could explain why CAPE treatment decreased irradiation-induced interstitial pneumonitis 3 and 12 weeks post-irradiation. Moreover, the results showed CAPE treatment is effective in reducing the expression of TGF-β1 after irradiation. TGF-β is a potent chemoattractant for fibroblasts and triggers the expression of extracellular matrix components in pulmonary fibrosis. The predominant localization of TGF-β in areas of inflammatory cell infiltrates and fibrosis suggests involvement of this cytokine in the pathogenesis of radiation- induced pulmonary fibrosis [32,33]. The mechanisms by which CAPE decreases TGF-β are unclear. It is probably through the NF-κB dependent pathway [34,35] or a process indirectly related to NF-κB [36,37]. In view of theses studies, we propose that CAPE treatment reduces radiation- induced pulmonary inflammation and fibrosis after a longer follow up time.

In summary, we have shown CAPE plays an important role in decreasing radiation pneumonitis by inflammatory cytokines, at least in part, without causing significant cytotoxicity. Based on this observation, CAPE is a promising adjuvant agent in the radiation treatment of lung cancer.

## Competing interests

The author(s) declare that they have no competing interests.

## Authors' contributions

MFC performed the study and drafted the manuscript. PCK conceived part of the study and performed the statistical analysis. PYL helped in histology and IHC staining. CTY and SKL conceived of the study and participated in its design and coordination.

WCC conceived of the study, participated in its design and coordination and assisted in editing of manuscript. All authors read and approved the final manuscript.

## Pre-publication history

The pre-publication history for this paper can be accessed here:



## References

[B1] Chiao C, Carothers AM, Grunberger D, Solomon G, Preston GA, Barrett JC (1995). Apoptosis and altered redox state induced by caffeic acid phenethyl ester (CAPE) in transformed rat fibroblast cells. Cancer Res.

[B2] Sud'ina GF, Mirzoeva OK, Pushkareva GA, Korshunova GA, Sumbatyan NV, Varfolomeev SD (1993). Caffeic acid phenethyl ester as a lipoxygenase inhibitor with antioxidant properties. FEBS Lett.

[B3] Bhimani RS, Troll W, Grunberger D, Frenkel K (1993). Inhibition of oxidative stress in Hela cells by chemopreventive agents. Cancer Res.

[B4] Chen YJ, Shiao MS, Wang SY (2001). The antioxidant caffeic acid phenethyl ester induces apoptosis associated with selective scavenging of hydrogen peroxide in human leukeic HL-60 cells. Anti-Cancer Drugs.

[B5] Chen MF, Wu CT, Chen YJ, Keng PC, Chen WC (2004). Cell killing and radiosensitization by caffeic acid phenethyl ester in lung cancer cells. J Radiat Res.

[B6] Huang MT, Ma W, Yen P, Xie JG, Han J, Frenkel K, Grunberger D, Conney AH (1996). Inhibitory effects of caffeic acid phenethyl ester (CAPE) on 12-O-tetradecanoylphorbol-13-acetate-induced tumor promotion in mouse skin and the synthesis of DNA, RNA and protein in HeLa cells. Carcinogenesis.

[B7] Hall EJ (2000). The Physics and chemistry of radiation absorption. Radiology for the radiologist.

[B8] Seed T, Kumar S, Whitnall M, Srinivasan V, Singh V, Elliott T, Landauer M, Miller A, Chang CM, Inal C, Deen J, Gehlhaus M, Jackson W, Hilyard E, Pendergrass J, Toles R, Villa V, Miner V, Stewart M, Benjack J, Danilenko D, Farrell C (2002). New strategies for the prevention of radiation injury: possible implications for countering radiation hazards. J Radiat Res.

[B9] Rubin P, Johnston CJ, Williams JP, McDonald S, Finkelstein JN (1995). A perpetual cascade of cytokines postirradiation leads to pulmonary fibrosis. Int J Radiat Oncol Biol Physics.

[B10] Hong JH, Chiang CS, Tsao CY, Lin PY, McBride WH, Wu CJ (1999). Rapid induction of cytokine gene expression in the lung after single and fractionated doses of radiation. Int Radiat Biol.

[B11] Michaluart P, Masferrer JL, Carothers AM, Subbaramaiah K, Zweifel BS, Koboldt C, Mestre JR, Grunberger D, Sacks PG, Tanabe T, Dannenberg AJ (1999). Inhibitory effects of caffeic acid phenethyl ester on the activity and expression of cyclooxygenase-2 in human oral epithelial cells and in a rat model of inflammation. Cancer Res.

[B12] Orban Z, Mitsiades N, Burke TR, Tsokos M, Chrousos GP (2000). Caffeic acid phenethyl ester induces leukocyte apoptosis, modulates nuclear transcription factor NF-κB and suppresses acute inflammation. Neuroimmunomodul.

[B13] Natarajan K, Singh S, Burke TR, Grunberger D, Aggarwal BB (1996). Caffeic acid phenethyl ester is a potent and specific inhibitor of activation of nuclear transcription factor NF-κB. Proc Natl Acad Sci USA.

[B14] Fitzpatrick LR, Wang J, Le T (2001). Caffeic acid phenethyl ester, an inhibitor of nuclear transcription factor NF-κB, attenuates bacterial peptidoglycan polysaccharide- induced colitis in rats. J Pharmacol Exp Ther.

[B15] Chen MF, Keng PC, Shau H, Wu CT, Hu YC, Liao SK, Chen WC Inhibition of lung tumor growth and augmentation of radiosensitivity by decreasing peroxiredoxin I expression. Int J Radiat Oncol Biol Physics.

[B16] Mirkovic N, Voehringer DW, Story MD, McConkey DJ, McDonnell TJ, Meyn RE (1997). Resistance to radiation- induced apoptosis Bcl-2- expressing cells is reversed by depleting cellular thiols. Oncogene.

[B17] Nardini M, Leonardi F, Scaccini C, Virgili F (2000). Modulation of ceramide- induced NF-κB binding activity and apoptotic response by caffeic acid in U937 cells: Comparsion with other antioxidants. Free Rad Biol Med.

[B18] Kucharczak J, Simmons MJ, Fan Y, G'eline C (2003). To be, or not to be: NF-κB is the answer – role of Rel/ NF-κB in the regulation of apoptosis. Oncogene.

[B19] Russo SM, Tepper JE, Baldwin AS, Liu R, Adams J, Elliott P, Cusack JC (2001). Enhancement of radiosensitivity by proteason inhibition: implication for a role of NF-κB. Int J Radiat Oncol Biol Physics.

[B20] de Ridder M, Verovski VN, Van den Berge DL, Sermeus AB, Monsaert C, Wauters N, Storme GA (2003). Lipid A radiosensitizes hypoxic EMT-6 tumor cells: role of the NF-κB signaling pathway. Int J Radiat Oncol Biol Physics.

[B21] Wang JF, Jerrells TR, Spitzer JJ (1996). Decrease production of reactive oxygen intermediates is an early event during in vitro apoptosis of rat thymocytes. Free Rad Biol Med.

[B22] Molls M, van Beuningen D, Scherer E, Streffer C, Trott KR (1991). Radiation injury of the lung: Experimental studies, observations after radiotherapy and total body irradiation prior to bone marrow transplantation. Radiopathology of organs and tissues.

[B23] Franko AJ, Sharplin J (1994). Development of fibrosis after lung irradiation in relation to inflammation and lung function in mouse strain prone to fibrosis. Radiat Res.

[B24] Neurath MF, Becker C, Barbulescu K (1998). NF-κB in immune and inflammatory responses in the gut. Gut.

[B25] Cuzzocrea S, Pisano B, Dugo L, Ianaro A, Patel NS, Caputi AP, Thiemermann C (2004). Tempol reduced the activation of nuclear factor-κB in acute inflammation. Free Radical Research.

[B26] Haase MG, Klawitter A, Geyer P, Alheit H, Baumann M, Kriegel TM, Kasper M, Baretton GB (2003). Sustained elevation of NF-κB DNA binding activity in radiation- induced lung damage in rats. Int J Radiat Biol.

[B27] Linard C, Marquette C, Mathieu J, Pennequin A, Clarencon D, Denis M (2004). Acute induction of inflammatory cytokine expression after γ- irradiation in the rat: effect of an NF-κB inhibitor. Int J Radiat Oncol Biol Physics.

[B28] Wang Y, Meng A, Lang H, Brown SA, Konopa JL, Kindy MS, Schmiedt RA, Thompson JS, Zhou D (2004). Activation of nuclear factor κB in vivo selectively protects the murine small intestine against ionizing radiation induced damage. Cancer Research.

[B29] Haddad JJ, Land SC (2001). Nuclear Factor-κB blockade attenuates but does not abrogate lipopolysaccharide-dependent tumor necrosis factor-alpha biosynthesis in alveolar epithelial cells. Biochem Biophys Res Commun.

[B30] McBride WH (1995). Cytokine cascades in late normal tissue radiation responses. Int J Radiat Oncol Biol Physics.

[B31] Kovacs EJ (1991). Fibrogenic cytokines: the role of immune mediators in the development of scar tissue. Immunology Today.

[B32] Rube CE, Uthe D, Schmid KW, Richter KD, Wessel J, Schuck A, Willich N, Rube C (2000). Dose-dependent induction of transforming growth factor β (TGF-β) in the lung tissue of fibrosis- prone mice after thoracic irradiation. Int J Radiat Oncol Biol Physics.

[B33] Sime PJ, Xing Z, Graham FL, Csaky KG, Gauldie J (1997). Adenovirus- mediated gene transfer of active transforming factor -β induces prolonged severe fibrosis in rat lung. J clin Invest.

[B34] Rameshwar P, Narayanan R, Qian J, Denny TN, Colon C, Gascon P (2000). NF-κB as a central mediator in the induction of TGF-β in monocytes from patients with idiopathic myelofibrosis: an inflammatory response beyond the real of hemeostasis. J of Immunology.

[B35] Lawrance IC, Wu F, Leite AZ, Willis J, West GA, Fiocchi C, Chakravarti S (2003). A murine model of chronic inflammation- induced intestinal fibrosis down- regulated by abtisense NF-Kappa B. Gastroenterlogy.

[B36] Warshamana GS, Corti M, Brody AR (2001). TNF-α, PDGF, and TGF-beta (1) expression by primary mouse bronchiolar alveolar epithelial and mesenchymal cells: TNF-α induces TGF-beta (1). Exp Mol Patho.

[B37] Liu JY, Brody AR (2001). Increased TGF-β1 in the lungs of asbestos-exposed rats and mice: reduced expression in TNF- alpha receptor knockout mice. J Environ Pathol Toxicol Oncol.

